# Using verbal autopsy to track epidemic dynamics: the case of HIV-related mortality in South Africa

**DOI:** 10.1186/1478-7954-9-46

**Published:** 2011-08-05

**Authors:** Peter Byass, Kathleen Kahn, Edward Fottrell, Paul Mee, Mark A Collinson, Stephen M Tollman

**Affiliations:** 1 MRC/Wits Rural Public Health and Health Transitions Research Unit (Agincourt), School of Public Health, Faculty of Health Sciences, University of the Witwatersrand, Johannesburg, South Africa; 2 Umeå Centre for Global Health Research, Department of Public Health and Clinical Medicine, Umeå University, Umeå, Sweden; 3 Centre for International Health and Development, Institute of Child Health, University College London, London, UK

## Abstract

**Background:**

Verbal autopsy (VA) has often been used for point estimates of cause-specific mortality, but seldom to characterize long-term changes in epidemic patterns. Monitoring emerging causes of death involves practitioners' developing perceptions of diseases and demands consistent methods and practices. Here we retrospectively analyze HIV-related mortality in South Africa, using physician and modeled interpretation.

**Methods:**

Between 1992 and 2005, 94% of 6,153 deaths which occurred in the Agincourt subdistrict had VAs completed, and coded by two physicians and the InterVA model. The physician causes of death were consolidated into a single consensus underlying cause per case, with an additional physician arbitrating where different diagnoses persisted. HIV-related mortality rates and proportions of deaths coded as HIV-related by individual physicians, physician consensus, and the InterVA model were compared over time.

**Results:**

Approximately 20% of deaths were HIV-related, ranging from early low levels to tenfold-higher later population rates (2.5 per 1,000 person-years). Rates were higher among children under 5 years and adults 20 to 64 years. Adult mortality shifted to older ages as the epidemic progressed, with a noticeable number of HIV-related deaths in the over-65 year age group latterly. Early InterVA results suggested slightly higher initial HIV-related mortality than physician consensus found. Overall, physician consensus and InterVA results characterized the epidemic very similarly. Individual physicians showed marked interobserver variation, with consensus findings generally reflecting slightly lower proportions of HIV-related deaths. Aggregated findings for first versus second physician did not differ appreciably.

**Conclusions:**

VA effectively detected a very significant epidemic of HIV-related mortality. Using either physicians or InterVA gave closely comparable findings regarding the epidemic. The consistency between two physician coders per case (from a pool of 14) suggests that double coding may be unnecessary, although the consensus rate of HIV-related mortality was approximately 8% lower than by individual physicians. Consistency within and between individual physicians, individual perceptions of epidemic dynamics, and the inherent consistency of models are important considerations here. The ability of the InterVA model to track a more than tenfold increase in HIV-related mortality over time suggests that finely tuned "local" versions of models for VA interpretation are not necessary.

## Background

Verbal autopsy (VA) has become a widely established approach for characterizing cause of death patterns in settings where individual deaths are not routinely certified as to cause, with a variety of methods being used for both interview and interpretation phases [1]. Most often, VA has been applied for particular times, or over relatively short periods, to obtain point estimates of cause-specific mortality. However, as archives of VA data accumulate over time, possibilities of studying epidemic dynamics using VA approaches emerge. This is of interest in terms of measuring potential newly emerging causes of death [2], as well as for monitoring the dynamics of epidemiological transition [3]. But it also raises new methodological challenges, for example around consistent interpretation of VA into causes of death over long periods of time and consequently around practitioners' developing perceptions of new situations. More generally, it raises the question of how effectively VA methods are able to detect newly emerging causes of death.

Over the past two decades, southern Africa has experienced a massive and rapidly developing epidemic of HIV infection and associated mortality [4-6]. However, large-scale modeled estimates provide a rather imperfect picture of the epidemic, given that most deaths in southern Africa are neither certified nor medically investigated [7]. Localized populations with intensive surveillance, such as member centers of the INDEPTH Network [8], provide opportunities to look at specific examples in detail [9-11], even if this may generate a subsequent debate as to generalizability. A number of studies elsewhere have established the validity of VA methods for attributing deaths to HIV/AIDS, particularly among adults [12-16]. Nevertheless, there remain some unresolved issues about how to best handle co-causes of mortality in cases of HIV-related death, and willingness to attribute deaths to HIV, whatever methods are used, may be influenced by nonmedical factors such as social stigmatization [17,18].

HIV-related deaths are complex to count, since HIV-positive individuals are frequently affected by other diseases as a result of being immunologically compromised, and it can be difficult from VA data, in the absence of HIV serology, to determine the relative significance of AIDS versus other diseases in the processes leading to death. The 10^th ^version of the International Classification of Diseases (ICD-10) uses codes B20 to B24 as underlying causes representing HIV/AIDS in combination with other disease categories (B20 infectious and parasitic diseases, B21 malignant neoplasms, B22 other diseases including wasting, B23 other conditions, and B24 nonspecific AIDS) [19]. However, differentiating probable HIV-related deaths detected by VA into these subcategories may not be easy to achieve, particularly where there is no explicit evidence of HIV positivity.

The ability to interpret any VA interview reliably depends on several factors, including the quality and detail of information on signs and symptoms provided by the informant. In settings where stigma is high around a particular cause of death - as is often the case for HIV - sensitive information may be withheld from the interviewer. Extent of nondisclosure is likely to vary as an epidemic develops, starting from minimal levels when key symptoms are not yet widely known by informants, and when physicians may also not yet be attuned to a particular diagnosis. As a significant epidemic such as HIV/AIDS develops, stigma is likely to rise, together with nondisclosure of relevant details. In a mature epidemic - particularly in the case of HIV as antiretroviral treatments are rolled out - nondisclosure may wane. These patterns may have significant effects on the outcomes of VA interpretation.

The Agincourt Health and Socio-Demographic Surveillance Site in the rural northeast of South Africa has been documenting a geographically-defined population (around 70,000 people in 2005) since 1992, including registering deaths and following those up with VA interviews [20]. The start of this surveillance in 1992 coincided with the early stages of the HIV epidemic (at least in terms of HIV-related mortality) in this area, and hence the accumulated VA data enable a methodological exploration as to how the epidemic evolved. Our primary aim is to characterize the epidemic of HIV-related mortality in this population, comparing both physician-interpreted causes of death and probabilistically modeled causes of death from the same VA interview material. As subsidiary aims, we investigate (1) approaches for handling common co-causes of HIV-related mortality, such as tuberculosis, malnutrition, and chronic gastroenteritis, and (2) variations between different coding physicians' responses to the emerging epidemic. Although this paper deals specifically with an epidemic of HIV-related mortality, findings are discussed in terms of using VA for monitoring long-term dynamics in mortality patterns.

## Methods

The analyses in this paper are based on the entire series of 6,153 deaths (among all ages) in the Agincourt population from 1992 to 2005, as previously described in terms of primary-care planning [21] and in a comparison between physician and modeled VA interpretation [22]. VA interviews were successfully completed for 5,794 deaths (94.2%), using a questionnaire developed before international standards were agreed upon. These VA interviews were subsequently coded by two independent physicians who attempted to reach consensus where their diagnoses differed, with a third reviewing and intervening in case of disagreement. If no consensus could be reached, the cause of death was recorded as "undetermined." During the period from 1992 to 2005, 14 physician reviewers were involved in VA interpretation during various subperiods. In 373 (6.4%) of VA reviews, it was not possible to trace the identities of the coding physicians. The InterVA model (http://www.interva.net) was also applied to the VA interview material, as described previously [22]. This public-domain model relates input indicators (history, signs, symptoms from VA interview material) to likely cause(s) of death using Bayesian probabilities. A standard grid of conditional prior probabilities was defined by an expert panel of physicians [23]. The model has subsequently been evaluated in a number of settings [22,24]. As a standard model designed for cause of death determination in low- and middle-income countries, it has the advantage of consistency over time and place [25].

A dataset was compiled (using Microsoft FoxPro) containing the two independent physician interpretations (main cause, possible immediate and contributing causes with ICD-10 codes), the physicians' consensus finding as to underlying cause (based primarily on the individual physicians' main cause findings), and the InterVA version 3.2 results (up to three likely causes per case, each associated with a quantified likelihood). The HIV level for the InterVA model was set to "high" and malaria set to "low," based on existing knowledge of causes of death in this population, as discussed previously [22]. The concept behind this setting in the InterVA model is analogous to a coding physician knowing that HIV or malaria represent more-common or less-common public health problems in a particular population, irrespective of the details around any individual death or detailed prior knowledge of cause-specific mortality. Age groups were defined as under 1 year, 1 to 4 years, 5 to 19 years, 20 to 49 years, 50 to 64 years, and 65 years and over. Analyses used Stata 10.

Surveillance-based studies in the Agincourt subdistrict were reviewed and approved by the Committee for Research on Human Subjects (Medical) of the University of the Witwatersrand, Johannesburg, South Africa (protocol M960720). Informed consent was obtained at the individual and household levels at every follow-up visit, whereas community consent from civic and traditional leadership was secured at the start of surveillance and reaffirmed from time to time. Feedback on cause of death patterns is presented to local communities and health service providers annually.

## Results

### The evolving epidemic of HIV-related mortality

**Figure 1:**
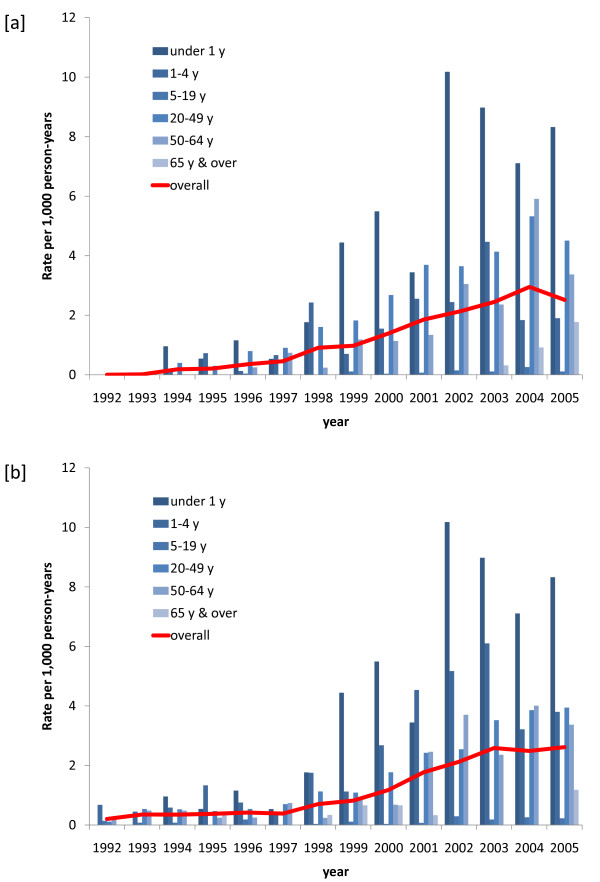
**HIV-specific mortality rates by age group by (a) physician consensus interpretation of VA data and (b) by InterVA interpretation (HIV-related death as most likely cause)**.

Figure 1 shows the evolution of HIV-related mortality, both overall and by age group, in the Agincourt population, calculated as the rates (per 1,000 person-years) of physician consensus underlying cause being coded as ICD-10 B20-B24 (1,136 deaths, 18.4%), or the rates of most likely cause from InterVA being HIV/AIDS-related death (1,146 deaths, 18.6%). Both approaches showed very similar patterns over time and within age groups, with a huge increase from no HIV-related deaths in 1992 to 2.5 per 1,000 person-years in 2005 according to physician coding, and correspondingly from 0.2 to 2.6 per 1,000 person-years according to InterVA. Table 1 shows numbers of deaths according to physicians and InterVA, by age, sex, and period.

**Table 1:** Characteristics of HIV-related deaths in Agincourt, South Africa, using VA data interpreted according to physician consensus on underlying cause and InterVA most likely cause

characteristic		period			
		
		1992-94	1995-97	1998-2001	2002-05
**physician consensus underlying cause = B20-B24 **(n = 1,136)					

total		13	69	357	697

sex	female	6	30	177	365
	
	male	7	39	180	332

age group	under 1 year	2	4	27	55
	
	1 to 4 years	1	12	52	71
	
	5 to 19 years	0	1	6	16
	
	20 to 49 years	10	48	255	480
	
	50 to 64 years	0	4	17	67
	
	65 years & over	0	0	0	8

mean age (SD)		27.5 (15.7)	30.2 (17.7)	27.6 (16.1)	30.9 (17.4)

					

**InterVA most likely cause = HIV-related **(n = 1,146)					

total		57	79	313	697

sex	female	34	42	177	425
	
	male	23	37	136	272

age group	under 1 year	5	7	41	96
	
	1 to 4 years	14	20	72	122
	
	5 to 19 years	7	5	7	26
	
	20 to 49 years	26	41	168	386
	
	50 to 64 years	5	5	19	63
	
	65 years & over	0	1	6	4

mean age (SD)		25.0 (20.6)	25.7 (20.8)	24.3 (19.8)	26.4 (19.7)

Only 63/6,153 (1.0%) of the overall VA records explicitly mentioned HIV positivity in the interview material, so the overwhelming majority of conclusions on HIV-related deaths both by the physicians and the model reflected circumstantial findings. When data for the period from 1992 to 1994 were rerun with InterVA set to "low" HIV, the number of cases most likely due to HIV-related causes decreased from 57 to eight out of a total of 707 deaths (8.1% to 1.1%). Physician consensus findings for the same period recorded 13 cases (1.8%), although a total of 20 cases (2.8%) were HIV-related according to at least one physician. However, among the 51 cases rated as HIV-related by the model ("high" setting) but not by physician consensus for this period, the most common underlying cause attributed by physicians was malnutrition (nine cases, 17.6%). By contrast, overall physician consensus results for 1992-1994 recorded 4.2% for malnutrition, compared with 1.3% for 1995-2005.

### Effects of different approaches for estimating HIV-related mortality

In addition to the physician consensus material on underlying causes of death that were identified as HIV-related, an additional 18 cases involved HIV as the physician consensus contributory cause. From this revised total of 1,154 HIV-related deaths, 693 (60.0%) were concluded in the physician consensus to have an infection (ICD B20), out of which 148 (12.7%) were specifically mentioned as tuberculosis. Ten cases (0.9%) had malignancies (B21), and 99 (8.6%) had chronic gastroenteritis or malnutrition (B22).

Using the alternative approach of the InterVA model, a total of 1,237 cases were rated as probably HIV-related, although in 91 of these HIV was not the most likely cause. Of the 1,237 cases, 156 (12.6%) were also identified as being associated with tuberculosis and 10 (0.8%) with other infections (B20), three (0.2%) with malignancies (B21), and 18 (1.5%) with chronic gastroenteritis or malnutrition (B22).

### Interphysician variations in attributing HIV-related mortality

**Figure 2:**
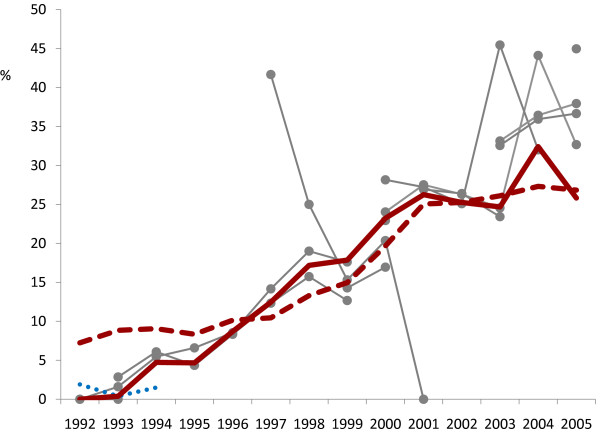
**Proportion of HIV-related deaths by year, according to physician consensus (heavy solid line) and opinions from 12 individual physicians who participated in coding during various periods (thin lines joining markers)**. The InterVA model results are represented by the heavy dashed line, with the alternative "low" HIV setting for 1992-1994 represented by the dotted line.

Of the 14 physicians coding this series of VAs, two completed very few (two and 16 cases respectively) and have been excluded from further consideration of interphysician variation. Of the 12 remaining, there were between two and five physicians coding VAs in any one year. No individual carried out work over the entire period. Figure 2 shows the overall proportions of physician consensus and InterVA HIV-related deaths by year, together with the proportions rated by the various physicians. In addition, the "low" HIV InterVA results for 1992-1994 are shown. Table 2 shows the proportions of HIV-related deaths as coded by first and second physician coders (irrespective of individual physician identity) compared with the revised physician consensus proportions, by year. The overall proportion of HIV-related mortality after achieving consensus was around 8% lower than single physician opinions (19.9% compared with 21.6%, ratio 0.92).

**Table 2:** HIV-related deaths (numbers and proportions) according to first and second physician coders and physician consensus, by year

year	deaths	HIV-related deaths (ICD codes B20-B24) n (%)		
		
		first physician	second physician	physician consensus
1992	152	0 (0)	0 (0)	0 (0)

1993	260	3 (1.2)	1 (0.4)	1 (0.4)

1994	254	15 (5.9)	12 (4.7)	12 (4.7)

1995	300	17 (5.7)	13 (4.3)	14 (4.7)

1996	276	25 (9.1)	24 (8.7)	24 (8.7)

1997	249	33 (13.3)	35 (14.1)	31 (12.4)

1998	361	59 (16.3)	66 (18.3)	62 (17.2)

1999	381	58 (15.2)	68 (17.8)	68 (17.8)

2000	422	97 (23.0)	96 (22.7)	98 (23.2)

2001	499	123 (24.6)	126 (25.3)	131 (26.3)

2002	594	137 (23.1)	148 (24.9)	150 (25.3)

2003	701	182 (26.0)	176 (25.1)	173 (24.7)

2004	648	237 (36.6)	236 (36.4)	210 (32.4)

2005	697	265 (38.0)	253 (36.3)	180 (25.8)

overall	5,794	1,251 (21.6)	1,254 (21.6)	1,154 (19.9)

## Discussion

It is clear that the progression of the epidemic of HIV-related mortality in this rural South African community, with population-based rates increasing more than tenfold over a 14-year period, was successfully detected and tracked by means of VA, in the absence of any more rigorous routine procedures for following up deaths and their causes. Although one might not argue for VA as the epidemiological method of choice for this purpose, the reality across much of the world is that there is no realistic alternative for the time being [26]. Even where deaths are supposed to be certified, there can be considerable difficulties in accurately capturing and recording deaths related to HIV/AIDS [27]. How VA material can best be interpreted into cause of death findings including HIV-related mortality is thus a very important issue, which can then form the basis of understandings of population health, for example patterns of social disparities [28].

The validity, reliability, and consistency with which VA data can be interpreted, particularly in terms of HIV-related mortality, are important issues. Both the physician-based and modeled approaches presented here yielded very similar results in terms of characterizing the epidemic. Intuitively plausible trends, such as the increasing age of HIV-related deaths observed as the epidemic developed (according to both approaches), presumably following developments in care and treatment, are encouraging. The InterVA model was not specifically designed to deliver ICD-10 codes, and so the major comparison here was equivalence at the B2* level rather than at the third digit level. As is usually the case where VA is used, there is no gold standard against which to absolutely compare these findings. Even if we knew the HIV serostatus for every death, there would still be difficulties in determining which deaths were actually attributable to HIV. However, it is very unlikely that the closely similar epidemic patterns shown for the two methods in Figure 1 would be similar entirely by chance, and in that sense both lend credence to the other. But, as we have noted previously [22], the physician approach was very time-consuming and expensive compared with probabilistic modelling, and the delays and expense involved in the physician process may be hard to justify from these results.

Since the "two physicians plus arbitrator" model of physician interpretation seems to have become a *de facto *(but not necessarily "gold") standard in much VA work, it is perhaps surprising that there have been few detailed analyses of individual physicians' opinions compared with physician consensus findings in VA studies using this method, with some exceptions [29,30]. It is also important in this context to remember that concurrent findings do not necessarily constitute "truth" [31]. In the particular setting of this epidemic, where the incidence of HIV-related deaths was changing at a rate that was not necessarily clear to physicians at the time, especially in the early stages of epidemic, it was particularly relevant to examine the ways in which individual physician interpreters responded to the changing situation, as well as the effect on consensus findings. It is also noteworthy that a relatively large number of individual physicians were involved in the process over the 14-year period; it would be surprising if this were not the case in most longer-term VA operations. It is worth noting that large studies using multiple physicians to interpret cause of death are difficult to interpret and understand if details about interobserver effects are not presented. It is also clear from the results in Figure 2 that, in general, consensus rates tended to be slightly lower than individual physician rates, particularly in the later years. This could have important implications in considering whether to use only a single coding physician per case, as has previously been suggested [32]. While there was generally good consistency between first and second physician findings (averaging over individual physicians) as shown in Table 2, the generally slightly lower rates of HIV-related mortality from the consensus process would probably result in slightly higher levels of "undetermined" cause of death in an all-cause analysis than might have resulted from using only a single physician coder.

Around the inception of this HIV-related mortality epidemic, the relationship between individual physicians, consensus results and the "low" and "high" HIV settings for the InterVA model are particularly interesting. The proportional differences in rates among the various approaches were greatest during the first three years, as is clear from Figure 2. Initial work on the InterVA model suggested that only causes likely to vary by an order of magnitude in terms of overall proportion needed to have an adjustment [23], with the crossover between "low" and "high" being at around 1% of total mortality. The "high" setting was therefore the appropriate one overall here. The analogous "setting" in physician coding is represented by a physician's awareness of how common HIV-related mortality is in a population, irrespective of the detailed circumstances of a particular case. Physician consensus rates gave the lowest measure of HIV in the early years, and it seems that in the uncertain early stages of the epidemic it was particularly difficult to achieve consensus, even though some deaths were considered as HIV-related by one physician. This supposition is indirectly supported by finding that the physicians' highest rates of malnutrition-related mortality were recorded during that period, probably representing a misclassification of deaths that were at least partly HIV-related. Thus the reality here is that the HIV-related mortality rates between 1992 and 1994 were probably somewhere in between the various estimates shown in Figure 2. Conversely, individual physicians recorded appreciably more HIV-related mortality in the later years, compared with both the consensus and modeled findings, possibly reflecting physicians' inflated views of HIV latterly. Additionally, nondisclosure of sensitive details in VA interviews at various stages of the epidemic may have compromised both the physicians' and model's findings. In the case of the model, it is important to note that the HIV rates over the period increased tenfold without any information being given to the model about a likely increase over time. This illustrates the relatively noncritical magnitudes of the cause-specific prior probabilities incorporated in the model, and supports the notion that a single model can be used for interpreting VA data over wide ranges of time and place, maximizing the benefits of consistency for comparative purposes over different settings.

## Conclusions

VA was clearly able to identify the emergence and growth of a very significant epidemic of HIV-related mortality in this population, and using either physicians or probabilistic modeling to derive cause of death findings gave closely similar results. The evidence suggests that physicians were perhaps a little slow to recognize the early stages of the epidemic, while the model (at least when set to expect a "high" level of HIV mortality) may have slightly overestimated initially. However, the fact that a numerically constant model was able to characterize a greater-than-tenfold increase in HIV-related mortality over time is an important demonstration of the relative robustness of probabilistic modeling for VA interpretation. This suggests that there is no need for finely tuned "local" versions of models for VA interpretation, the proliferation of which would detract from the comparability of results over time and place.

## Competing interests

The authors declare that they have no competing interests.

## Authors' contributions

PB conceived the study, analyzed data, and drafted the manuscript. All authors contributed to critical reviews and developments in the paper. KK established and managed the VA system, and KK and SMT were involved in physician interpretation and arbitration of VA material. EF, PM, and MAC managed data and quality control. All authors read and approved the final manuscript.

## References

[B1] Fottrell E Byass P Verbal Autopsy - methods in transition Epidemiologic Reviews 2010 32 38 55 10.1093/epirev/mxq003 20203105

[B2] Nsubuga P Nwanyanwu O Nkengasong JN Mukanga D Trostle M Strengthening public health surveillance and response using the health systems strengthening agenda in developing countries BMC Public Health 2010 10 Suppl 1 S5 10.1186/1471-2458-10-S1-S5 21143827 PMC3005577

[B3] Karar ZA Alam N Streatfield PK Epidemiological transition in rural Bangladesh, 1986-2006 Global Health Action 2009 2 10.3402/gha.v2i0.1904 PMC2779938 20027273

[B4] Joint United Nations Program on HIV/AIDS Global report: UNAIDS report on the global AIDS epidemic 2010 2010 Geneva: UNAIDS http://www.unaids.org/en/media/unaids/contentassets/documents/unaidspublication/2010/20101123_globalreport_en.pdf ISBN 978-92-9173-871-7

[B5] Dorrington RE Johnson LF Bradshaw D Daniel T The Demographic Impact of HIV/AIDS in South Africa. National and Provincial Indicators for 2006 2006 Cape Town: Centre for Actuarial Research, South African Medical Research Council and Actuarial Society of South Africa http://www.mrc.ac.za/bod/DemographicImpactHIVIndicators.pdf

[B6] Bradshaw D Nannan N Groenewald P Joubert J Laubscher R Nojilana B Norman R Pieterse D Schneider M Provincial mortality in South Africa, 2000 - priority-setting for now and a benchmark for the future South African Medical Journal 2005 95 496 503 16156448

[B7] Byass P The Imperfect World of Global Health Estimates PLoS Medicine 2010 7 e1001006 10.1371/journal.pmed.1001006 21152416 PMC2994666

[B8] Bangha M Diagne A Bawah A Sankoh O Monitoring the Millennium Development Goals: the potential role of the INDEPTH Network Global Health Action 2010 3 5517 10.3402/gha.v3i0.5517 PMC2938980 20842216

[B9] Kanjala C Alberts M Byass P Burger S Spatial and temporal clustering of mortality in Digkale HDSS in rural northern South Africa Global Health Action 2010 Supp 1 10.3402/gha.v3i0.5236 PMC2935922 20838631

[B10] Hosegood V Vanneste A-M Timæus IM Levels and causes of adult mortality in rural SouthAfrica: the impact of AIDS AIDS 2004 18 663 671 10.1097/00002030-200403050-00011 15090772

[B11] Garrib A Jaffar S Knight S Bradshaw D Bennish ML Rates and causes of child mortality in an area of high HIV prevalence in rural South Africa Tropical Medicine and International Health 2006 11 1841 1848 10.1111/j.1365-3156.2006.01738.x 17176349

[B12] Chandramohan D Maude GH Rodrigues LC Hayes RJ Verbal autopsies for adult deaths: their development and validation in a multicentre study Tropical Medicine and International Health 1998 3 436 446 10.1046/j.1365-3156.1998.00255.x 9657505

[B13] Doctor HV Weinrebb AA Estimation of AIDS adult mortality by verbal autopsy in rural Malawi AIDS 2003 17 2509 2513 10.1097/00002030-200311210-00014 14600523

[B14] Lopman B Cook A Smith J Chawira G Urassa M Kumogola Y Isingo R Ihekweazu C Ruwende J Ndege M Gregson S Zaba B Boerma T Verbal autopsy can consistently measure AIDS mortality: a validation study in Tanzania and Zimbabwe Journal of Epidemiology and Community Health 2010 64 330e334 10.1136/jech.2008.081554 PMC2922698 19854751

[B15] Quigley MA Chandramohan D Rodrigues LC Diagnostic accuracy of physician review, expert algorithms and data-derived algorithms in adult verbal autopsies International Journal of Epidemiology 1999 28 1081 1087 10.1093/ije/28.6.1081 10661651

[B16] Tensou B Araya T Telake DS Byass P Berhane Y Kebebew T Sanders EJ Reniers G Evaluating the InterVA model for determining AIDS mortality from verbal autopsies in the adult population of Addis Ababa Tropical Medicine and International Health 2010 15 547 553 20214760 10.1111/j.1365-3156.2010.02484.x PMC3901008

[B17] Groenewald P Nannan N Bourne D Laubscher R Bradshaw D Identifying deaths from AIDS in South Africa AIDS 2005 19 193 201 10.1097/00002030-200501280-00012 15668545

[B18] Blacker J The impact of AIDS on adult mortality: evidence from national and regional statistics AIDS 2004 18 suppl 2 S19 S26 15319740 10.1097/00002030-200406002-00003

[B19] World Health Organization ICD-10: international statistical classification of diseases and related health problems: tenth revision - 2nd edition, volume 2 2004 Geneva: World Health Organization http://www.who.int/classifications/icd/ICD-10_2nd_ed_volume2.pdf ISBN 92 4 154653 0

[B20] Kahn K Tollman SM Collinson MA Clark SJ Twine R Clark BD Shabangu M Gomez-Olive FX Mokoena O Garenne ML Research into health, population, and social transitions in rural South Africa: Data and methods of the Agincourt Health and Demographic Surveillance System Scandinavian Journal of Public Health 2007 35 supplement 69 8 20 10.1080/14034950701505031 PMC2826136 17676498

[B21] Tollman SM Kahn K Sartorius B Collinson MA Clark SJ Garenne ML Implications of mortality transition for primary health care in rural South Africa: a population-based surveillance study Lancet 2008 372 893 901 10.1016/S0140-6736(08)61399-9 18790312 PMC2602585

[B22] Byass P Kahn K Fottrell E Collinson MA Tollman SM Moving from Data on Deaths to Public Health Policy in Agincourt, South Africa: Approaches to Analysing and Understanding Verbal Autopsy Findings PLoS Medicine 2010 7 e1000325 10.1371/journal.pmed.1000325 20808956 PMC2923087

[B23] Byass P Fottrell E Huong DL Berhane Y Corrah T Kahn K Muhe L Van DD Refining a probabilistic model for interpreting verbal autopsy data Scandinavian Journal of Public Health 2006 34 26 31 10.1080/14034940510032202 16449041 PMC2833983

[B24] Fantahun M Fottrell E Berhane Y Wall S Hogberg U Byass P Assessing a new approach to verbal autopsy interpretation in a rural Ethiopian community: the InterVA model Bulletin of the World Health Organization 2006 84 204 210 10.2471/BLT.05.028712 16583079 PMC2627286

[B25] Fottrell E Kahn K Ng N Sartorius B Huong DL Minh HV Fantahun M Byass P Mortality measurement in transition: proof of principle for standardised multi-country comparisons Tropical Medicine and International Health 2010 15 1256 1265 10.1111/j.1365-3156.2010.02601.x 20701726 PMC3085122

[B26] Garenne M Fauveau V Potential and limits of verbal autopsies Bulletin of the World Health Organization 2006 84 164 10.2471/BLT.05.029124 16583068 PMC2627293

[B27] Nojilana B Groenewald P Bradshaw D Reagon G Quality of cause of death certification at an academic hospital in Cape Town, South Africa South African Medical Journal 2009 99 648 652 20073291

[B28] Groenewald P Bradshaw D Daniels J Zinyakatira N Matzopoulos R Bourne D Shaikh N Naledi T Local-level mortality surveillance in resource-limited settings: a case study of Cape Town highlights disparities in health Bulletin of the World Health Organization 2010 88 444 451 20539858 10.2471/BLT.09.069435 PMC2878147

[B29] Morris SK Bassani DG Kumar R Awasthi S Paul VK Jha P Factors Associated with Physician Agreement on Verbal Autopsy of over 27000 Childhood Deaths in India PLoS ONE 2010 5 3 e9583 10.1371/journal.pone.0009583 20221398 PMC2833201

[B30] Khademi H Etemadi A Kamangar F Nouraie M Shakeri R Abaie B Pourshams A Bagheri M Hooshyar A Islami F Abnet CC Pharoah P Brennan P Boffetta P Dawsey SM Malekzadeh R Verbal Autopsy: Reliability and Validity Estimates for Causes of Death in the Golestan Cohort Study in Iran PLoS ONE 2010 5 6 e11183 10.1371/journal.pone.0011183 20567597 PMC2887437

[B31] Byass P The democratic fallacy in matters of clinical opinion: implications for analysing cause-of-death data Emerging Themes in Epidemiology 2011 8 1 10.1186/1742-7622-8-1 21223568 PMC3026021

[B32] Joshi R Lopez AD MacMahon S Reddy S Dandona R Dandona L Neal B Verbal autopsy coding: are multiple coders better than one? Bulletin of the World Health Organization 2009 87 51 57 10.2471/BLT.08.051250 19197404 PMC2649601

